# Catching Chances: The Movement to Be on the Ground and Research Ready before an Outbreak

**DOI:** 10.3390/v10080439

**Published:** 2018-08-19

**Authors:** David Brett-Major, James Lawler

**Affiliations:** 1 Department of Preventive Medicine and Biostatistics, F. Edward Hébert School of Medicine, Uniformed Services University, Bethesda, MD 20814, USA; 2 Global Center for Health Security, University of Nebraska Medical Center, Omaha, NE 68198, USA; james.lawler@unmc.edu

**Keywords:** filovirus, emergency clinical management research, medical countermeasures review, global health

## Abstract

After more than 28,000 Ebola virus disease cases and at least 11,000 deaths in West Africa during the 2014–2016 epidemic, the world remains without a licensed vaccine or therapeutic broadly available and demonstrated to alleviate suffering. This deficiency has been felt acutely in the two, short, following years with two Ebola virus outbreaks in the Democratic Republic of Congo (DRC), and a Marburg virus outbreak in Uganda. Despite billions of U.S. dollars invested in developing medical countermeasures for filoviruses in the antecedent decades, resulting in an array of preventative, diagnostic, and therapeutic products, none are available on commercial shelves. This paper explores why just-in-time research efforts in the field during the West Africa epidemic failed, as well as some recent initiatives to prevent similarly lost opportunities.

## 1. Where We Are Today

Tragically, despite more than 28,000 Ebola virus disease (EVD) cases and at least 11,000 deaths in West Africa during the 2014–2016 epidemic, the world remains without a licensed vaccine or therapeutic broadly available and demonstrated to alleviate suffering [1]. In the two years following the declared end of the outbreak in West Africa, Africa has experienced two Ebola virus outbreaks in the Democratic Republic of Congo (DRC), and a Marburg virus outbreak in Uganda. During the 2018 EVD outbreak in Equateur Province, DRC, a ring vaccination campaign was undertaken within the framework of compassionate use/expanded access [2]. While more than 3300 people were vaccinated, the vaccination campaign was not begun until after 31 of 38 confirmed cases had been identified, and the contribution of data from vaccinees toward safety and efficacy profiles is yet to be determined. In the past 25 years, the U.S. Government alone has spent billions of dollars on research and development for an array of preventative, diagnostic, and therapeutic countermeasures for filoviruses and other high consequence threats [3]. Nevertheless, commercial shelves remain conspicuously bare of these anti-filovirus products even though some of them sat unlicensed for nearly a decade prior to the West Africa EVD epidemic, such as the rVSZ-EBOV product eventually fielded for postexposure prophylaxis [4]. While there are a myriad of causes of this including waxing and waning funding cycles and other aspects of basic and translational science management, this paper explores why just-in-time field research efforts in the epidemic failed, as well as some recent initiatives to prevent similarly lost opportunities. It is about field clinical trial processes and their performance.

Experimental products appeared relatively late in the field during the 2014–2016 EVD epidemic, and only a few found their way into what could be considered clinical trials. The Zaire-specific monoclonal antibody cocktail ZMapp, in particular, was administered in a randomized trial predominantly in Sierra Leone, Guinea, and Liberia, in a setting where other products also were used [5]. The small molecule, direct acting antiviral drug favipiravir was assessed in Guinea [6,7]. The small inhibitory RNA drug TKM-130803 was evaluated in Sierra Leone [8]. And, at the close of the epidemic, the small molecule, direct acting antiviral drug GS-5734 entered evaluation as a method of clearing persistent virus in sanctuary body compartments like the testicles [9]. And, yet, while ZMapp is available in a small expanded access program for compassionate use, and others are joining that list, the ultimate role of these therapeutics remains uncertain. Vaccines fared a little bit better in the process. A recombinant, replication-competent vesicular stomatitis virus-based vaccine (rVZV-ZEBOV) appeared to be effective in postexposure prophylaxis in Guinea [10]. While not licensed, it is being used in outbreaks such as this year’s in DRC. The longevity of its immunity is uncertain, and other vaccines continue to be evaluated [11,12]. The most advanced countermeasure in terms of availability resulted from technical activity during the epidemic, a lateral flow immunoassay for the virus which has an emergency use authorization [13].

Why, then, did all of this effort in discovery, development, and testing not yield available products after so much human and community impact? The reasons are less complicated than one new to the subject might suspect: biology, timing, readiness, and understanding.

### 1.1. Biology

Soon after the U.S. Government dramatically expanded its civilian biodefense enterprise in the aftermath of the September 11th and anthrax letter events, policy makers surmised that conventional human trial clinical data normally required for Food and Drug Administration (FDA) licensure would be difficult to acquire for diseases caused by threat pathogens that were uncommonly encountered in the US or other developed world settings. In response, the FDA created new regulations in 2002 known as the “Animal Rule” (21 CFR 314.600-650; 21 CFR 601.90-95). Although, technically, 17 product uses have been approved through the Animal Rule since 2003, only three represent first-licensed, truly novel products [14]. Two are treatments for inhalational anthrax and are monoclonal antibodies; and, the most recent is a small molecule therapy for smallpox [15]. Why has the biodefense and public health emergency enterprise failed in licensing more novel products under the animal rule? The reasons are manifold, but the most basic of them is biology. In the interval years since the advent of the Animal Rule, scientists have come to better appreciate the limitations of animal models of complex host–disease phenomena that are critical to understanding safety and efficacy of therapeutics and vaccines [16]. As a result, the FDA may be reluctant to base determinations solely on pre-clinical and animal data without at least supporting human clinical data—and this message has been clearly communicated within the filovirus therapeutic R&D community [17]. This stance threw a wrench into R&D pipelines that had been built specifically to exploit the Animal Rule, and government agencies and their biotech/pharmaceutical partners responsible for filovirus and other biodefense related product development were not prepared to acquire human clinical data. Notably validating such skepticism of over-reliance on animal models was a single-arm clinical trial of an experimental small interfering RNA therapeutic during the West Africa epidemic [8]. Although this product had performed remarkably well in nonhuman primate models, patients who received it in West Africa experienced 79% case fatality—considerably higher than the expected background CFR in treatment units at the time. While the enrolled patients were very ill and the product is not conclusively the cause of the observed mortality, this study clearly demonstrated dichotomous outcomes between animal model and human clinical experience.

### 1.2. Timing

In general, meaningful efficacy is easier to demonstrate than safety. The paradigm of medical therapeutics is built upon a relatively high expectation for therapeutic benefit and very low tolerance for adverse effects—reflecting the fundamental Hippocratic principle of primum non nocere. By the time experimental products reach clinical trials, among 100 patients investigators might expect therapeutic benefit in 30 or more, but they would anticipate a very small number of them to be hurt by the product. This creates the condition where detection of important safety signals requires large and time-consuming trials. Consider the experience with the pain reliever rofexocib [18,19]. Very large studies with thousands of patients showed efficacy, but recognition of increased myocardial infarction rates in at risk patients took tens of thousands of patients, actually revealing a broad effect of related medications. Rare, severe illness disease events result in disjointed opportunities to test a therapeutic, and noise from the consequence of severe illness create the challenge of discerning whether or not adverse events are attributable to the investigational product. The ability to discern efficacy, too, is challenged by this dynamic. If current application of the FDA Animal Rule requires at least some validating human clinical data, confidence regarding correlating signals around efficacy, and especially, safety is directly proportional to the number of patients that can be enrolled. As in any statistical analysis driven process, numbers matter. While large filovirus disease outbreaks sometimes occur, most events over time are relatively small with short times to peak case counts [20]. Consequently, a research effort must enroll participants early in an event, and repeatedly across events.

### 1.3. Readiness

In order to take advantage of the small window of time available to acquire meaningful clinical data in outbreaks, a research team and its many stakeholders must be ready; ready not only to affect research operations with little warning, but to do so in a way that minimizes the impact of heterogeneity of populations, communities, emergency response, and clinical care that often accompanies rare, sporadic events.

Consider the many factors important to a successful evaluation of a therapeutic product in an emergency.

- A research plan tailored to a product or products with an expectation of value to the patient, that is executable in a low-resource environment and allows useful analytic assessment of data
- Plan acceptance and participation by industry, funding, regulatory, emergency management, and public health entities at home, at the location(s) of the emergency—including the local population writ large—and often in the international community
- Designated and practiced administrative and financial resources that allow rapid movement, implementation, and sustainment of research efforts [21]
- Manpower that is trained, equipped, supplied, and available for safe and effective care of high consequence pathogen patients, incorporating case management and laboratory needs, environmental and hygiene services, logistics, and in some instances security [22,23,24]
- A patient-centered outcome strategy that mitigates differences between events through early suspected case identification, ready access to care, and high quality clinical intervention with or without the investigational therapeutic [25]
- An integration strategy that at best mutually reinforces and at worst de-conflicts tandem emergency response and investigational drug evaluation efforts
- Developed and practiced procedures for the entire team in good clinical practice and research execution in a high consequence pathogen environment, with reinforcing systems, monitoring, assessment, and intervention to ensure applicable, high quality data
- Transition strategies for study participants as they are discharged into the at-large case management stream, as well as for the study itself exploring longer term outcomes and contributing to survivor consequence management and assessment
- Sustainment strategies for stakeholder update on progress, redirection as needed, and sufficient resourcing to carry the effort through the course of each emergency
- Reintegration strategies for manpower and other resources
- A clear approach to process and impact observation, reflection, course correction, and generation of next steps

Each of the ad hoc therapeutic trial efforts in the West Africa epidemic attempted to create some of these conditions. None of them achieved a majority. In fairness, however, no one was ready beforehand, and attempting to achieve these goals in the middle of an emergency is an impossible task. None of the readiness conditions were met by anyone when the epidemic started, nor when it was recognized as an epidemic, nor even when people started accepting that clinical research might be a valid pursuit in it. All of the efforts began late, and in most cases after the peak of the epidemic in their respective locations.

### 1.4. Understanding

In order to be ready and act in an effective, timely manner, stakeholders must have a common understanding of what is to be accomplished. First steps to this are a common operating picture and joint planning process. When the West Africa epidemic was declared a public health emergency of international concern (PHEIC) by the Director General of the World Health Organization (WHO), WHO established a new metrics monitoring and situational report process that broadly displayed its own operating picture, including a dashboard [26]. Other agencies and actors also did this, most notably the U.S. Centers for Disease Control and Prevention (CDC), Médecins Sans Frontières (MSF), and UNICEF. Understandably, each display reflected that individual entity’s interests and needs for social and resource mobilization—not necessarily a collective picture. This practice continues. One must simply read a WHO situational report of this summer’s DRC outbreaks, in particular the case management section, and see the sparse mention of MSF—the primary clinical care provider in this instance—or for that matter, any detailed metrics around patient outcomes, to see that the displays are not shared and the operating picture is not common [27]. Centralized communication means sacrificing some aspect of control, and every entity has its own emergency management interests [28]. But, the distinct displays of individual understanding reflected also the partitioned way that each group undertook its interests in other aspects of the response. Coordination meetings occurred, but clinical trial activity was competitive, as happens in the usual course of field research, and in some instances partners maneuvered sharply to exclude or co-opt activity within shifting zones of influence.

These challenges impact conversations even in areas where diverse groups are working hard on cooperation. Arriving at a common understanding of the interests to be met by a cooperative activity is difficult when conversations are based upon position and not interests. Fully exposing interests can be tricky for a coordinating or implementing entity seeking funding, as that can deter funders who want their money applied towards what they perceive to be their specific interests. Innovators want an idea to be adopted by a funded development partner. Governments want a product in their stockpile. Industry wants a licensed drug with a market at acceptable delivery cost. MSF and other civil society and intergovernmental agencies want to maintain community access for responses so that outbreaks are interrupted quickly enough for mobilized resources to be within scope. Researchers want study success. Healthcare and other workers related to an effort want to be helpful, but also do not want to become infected, to be rejected by their community, or to be undervalued. Communities want their own afflicted persons to receive care while also not wanting to have Ebola virus in their midst. Patients want to suffer less, not die, and have a future post-emergency. Everyone wants to be seen being of value, and wants to be what they perceive as properly resourced.

When these interests are not openly discussed in a manner seeking agreed remedies, strange hybrid solutions result built upon preconceived notions of what everyone else is thinking. This resulted in the convenience sample study approaches to therapeutic interventions in 2014–2016, with variable incorporation of usual clinical care metrics and biomarkers in critical care research, such as electrolytes and volume of fluid received, and variable assertions regarding what might be a useful product to use. Clinical research lessons learned stemming from funders and policy groups have put forward what might be common requirements if not common interests, but have not yet embraced these baser aspects of understanding [29].

Optimal conversations take time and effort, but the results can be worth the effort. Lessons can be taken from environmental dispute resolution, where diverse stakeholders often have starkly opposing views of what should occur in situations near homes and investments. It poses a familiar multi-party, geospatial landscape to health emergencies. “Communities assume that when a project is proposed that the agencies overseeing the project will coordinate. They also assume that the coordination will result in less disruption to a community. However, this is not always true. Sometimes agencies do not coordinate with each other and communities are subjected to a series of disjointed projects” [30]. But, consensus approaches relying upon stepwise issue identification, exploration, then assessment and resolution are time intensive. One notable consent decree resulting in a highway overpass in southern California was the result of years of very active engagement. In the operational public health environment, there are additional challenges in this kind of discussion related to assessing current capacity in an affected community, and whether or not that capacity will be durably improved by the emergency response.

No wonder, then, that all parties were poorly footed to do research comprehensively and well in 2014–2016. Given the heavy work to achieve good timing, readiness, and understanding for actions in a health emergency, initiating the clinical research effort in advance, aggressively and durably, is the only solution.

## 2. Hope for Tomorrow

Most stakeholders are not funded to do either the holistic or detailed work required in inter-emergency periods. Nonetheless, there are evolving attempts to be ready that collectively may yield benefits if they are able to continue and develop. These efforts replace the older model of fielding a research team on call and praying for access with response partners that yielded the outputs from the West Africa EVD epidemic and other health emergencies. Structurally, each of these new efforts can be categorized as some combination of what we will call embedded deployment, organic, or network-based. We discuss these various models as potential hope for the future.

### 2.1. Embedded Deployment

In this structure, deploying entities which provide care as part of the response effort to health emergencies from high consequence pathogens develop and employ an internal research capability, sometimes augmented by collaborators. Two examples of this approach are the work of Médecins Sans Frontières (MSF) and International Medical Corps (IMC). MSF is a storied responder to both filovirus and cholera outbreaks. For decades, they have been the sole principal civil society actor to do so for filovirus outbreaks, only joined by others in the setting of the overwhelming caseload in the West Africa epidemic. MSF has managed its own ethics review board since 2002, and maintains a broad research portfolio [31,32]. In the Ebola epidemic, MSF research activity centered upon a cooperation fielding favipiravir [6,7,33,34]. IMC, similarly, has developed organic research programming [35]. In the epidemic, its Liberia site participated in the assessment of ZMapp, as well as various nonpharmacologic observational research such as patient monitoring [5,36,37,38].

Advantages of this approach include ready access to patients of interest, existing operational activities and logistics, predesignated scientific leadership, as well as monitoring and assessment mechanisms. Well-executed, this approach also presents opportunities to ensure higher quality patient management and laboratory services when the research is being conducted, as the entity doing the research also is responsible for care. Often, basic clinical laboratory services useful for care of the patient are not available in usual response settings. Unfortunately, these potential advantages did not make the research response in the early summer 2018 Ebola outbreak in DRC immune to the following, evident disadvantages. While an investigational vaccine, the rVSV product, was fielded, more robust therapeutic agent research did not occur despite a fairly large caseload. The disadvantages include lags in initiating the research which result from not yet having completed specific local tasks such as navigating local regulatory processes, and an inability to build community acceptance of research before it is needed. Time will tell how performance evolves in the summer’s second DRC Ebola emergency.

### 2.2. Organic

In early 2015, US Department of Defense research institute partners convened a working group to overcome the disadvantages experienced in previous rapid response research attempts. Labeled the Joint Mobile Emerging Disease Intervention Clinical Capability (JMEDICC), it ultimately initiated a sepsis clinical trial capability in western Uganda, an area of high filovirus outbreak risk [39]. JMEDICC is designed to shift its work to filovirus therapeutic clinical trial activity in the event of an emergency. It has a hub and spoke structure, based at Fort Portal Regional Referral Hospital and with an ability to move work to an emergency response location as a science module to the clinical effort. This strategy allows continual use of clinical research and severely ill patient management competencies at the hub while also developing and maintaining high consequence pathogen management skills at the hub and, when drilled or called, spoke sites. International partners assist with training, monitoring, assessment, and when needed, augmentation.

The principal advantage of the organic approach is the ongoing circle of readiness and trust built with the participating partners, which facilitate pre-evaluated clinical trial protocols, established patterns of cooperation between clinical and public health partners, and health services and emergency partners. Additionally, a focus on care quality is more readily accessible as planning for care and research happen conjointly with a particular context. The hub-spoke concept seeks to mitigate the disadvantage of having invested to site the capability into a specific subregion that may or may not experience the sought emergency event. Perhaps ironically, filovirus events have continued to occur nearby as JMEDICC has developed. Also, funding structures generally are not aligned for sustainment of this approach, as the requisite clinical competencies, research activity, and response preparation often are sourced from very separate funding lines that rarely converge for strategic purposes.

While not fully fitting this model, the National Institute of Allergy and Infectious Diseases (NIAID) took steps in this direction in Liberia. In the PREVAIL series of trials, NIAID continued some research capability established for vaccine trials and assessment of ZMapp during the Ebola epidemic [5,12]. It has added and absorbed Ebola survivor studies, including a trial of GS-5734 to eradicate persistent virus in immunologic sanctuary sites [40]. This effort remains largely unlinked from care provision activities. However, it may be well placed to merge with clinical management response efforts should they be required where it is established. 

### 2.3. Network-Based

The European Commission has funded the PREPARE project to ready infectious diseases clinical trial units for the conduct of large, harmonized trials in an emergency [41]. The work is in its early days, but it exploits established academic centers. How the network will interface with isolation care in the high consequence pathogen setting is not yet clear. Some of the European partners maintain an isolation care capability in their institutions. However, the program’s participating hospital-based clinical research networks lack a critical care focus, and work in antimicrobial resistance, for instance, may or may not be compatible to requirements in the careful management and research assessment of severely ill patients with a high consequence pathogen. Its scope is ambitious, incorporating aspects of education and manpower development as well as diagnostics testing. Their work has not yet seemed to focus on ill patients. Some members of the PREPARE project are exploring ways to incorporate African and other at risk community health care sites into their strategy. PREVAIL has a more focused approach with a continued networking strategy with U.S. and European isolation care centers regarding therapeutic product assessment.

Network approaches seek to balance the application of resources with enhancing opportunities to capture trial participants of interest. Broad geographic scope is an advantage of this approach, as well as the potential for pre-approved human use research regulatory pathways among well-resourced partners, in particular. There is risk in these approaches of placing time, money, and human resources too diffusely to execute complicated work. This can be mitigated by focusing intended effort, and building and practicing mechanisms for resources across the network to be applied in specific emergency research events. In each of these approaches, prospective research on field clinical trial processes and performance would be of value.

## 3. Summary and Next Steps

There are no perfect solutions to being ready to trial experimental medical countermeasures against high consequence pathogens as needed, at the beginning of an emergency wherever, and in whichever context it might occur. Planning must incorporate biology, timing, readiness, and understanding in practical ways. Investment must seek to develop and sustain useful aspects of the embedded deployment, organic, and network-based approaches. And, it must do so in ways that allow necessary flexibility to execute both safe and effective care of severely ill patients and mature research, while being meaningfully connected to the emergency risk management process.
